# Patients’ and healthcare providers’ perceptions of a mobile portal application for hospitalized patients

**DOI:** 10.1186/s12911-016-0363-7

**Published:** 2016-09-21

**Authors:** Kevin J. O’Leary, Rashmi K. Sharma, Audrey Killarney, Lyndsey S. O’Hara, Mary E. Lohman, Eckford Culver, David M. Liebovitz, Kenzie A. Cameron

**Affiliations:** 1 Division of Hospital Medicine, Northwestern University Feinberg School of Medicine, 211 E. Ontario Street, Suite 211, Chicago, IL 60611 USA; 2 Division of General Internal Medicine, University of Washington School of Medicine, Seattle, WA USA; 3 Northwestern Memorial HealthCare, Chicago, IL USA; 4 Division of General Internal Medicine, University of Chicago Medicine, Chicago, IL USA; 5 Division of General Internal Medicine and Geriatrics, Northwestern University Feinberg School of Medicine, Chicago, IL USA

**Keywords:** Patient-centered care, Patient portal, Personal health record, Hospitalized patient, Patient engagement

## Abstract

**Background:**

Hospital-based patient portals have the potential to better inform and engage patients in their care. We sought to assess patients’ and healthcare providers’ perceptions of a hospital-based portal and identify opportunities for design enhancements.

**Methods:**

We developed a mobile patient portal application including information about the care team, scheduled tests and procedures, and a list of active medications. Patients were offered use of tablet computers, with the portal application, during their hospitalization. We conducted semi-structured interviews of patients and provider focus groups. Text from transcribed interviews and focus groups was independently coded by two investigators using a constant comparative approach. Codes were reviewed by a third investigator and discrepancies resolved via consensus.

**Results:**

Overall, 18 patients completed semi-structured interviews and 21 providers participated in three focus groups. Patients found information provided by the portal to be useful, especially regarding team members and medications. Many patients described frequent use of games and non-clinical applications and felt the tablet helped them cope with their acute illness. Patients expressed a desire for additional detail about medications, test results, and the ability to record questions. Providers felt the portal improved patient engagement, but worried that additional features might result in a volume and complexity of information that could be overwhelming for patients. Providers also expressed concern over an enhanced portal’s impact on patient-provider communication and workflow.

**Conclusions:**

Optimizing a hospital-based patient portal will require attention to type, timing and format of information provided, as well as the impact on patient-provider communication and workflow.

**Electronic supplementary material:**

The online version of this article (doi:10.1186/s12911-016-0363-7) contains supplementary material, which is available to authorized users.

## Background

Hospital settings present important challenges to communication of medical information between providers and patients. Patients often have multiple active conditions, undergo numerous tests, and receive complex treatments that evolve throughout their hospital stay. Care teams are large and team membership is dynamic because of the need to provide care 24 h a day, 7 days a week [1–3]. Further, the verbal information provided to patients during daily rounds is seldom complemented by any other mode of communication [4]. As a result of these challenges, hospitalized patients often have incomplete comprehension and less than optimal engagement in their care [5–8].

Patient portals, now commonly used in ambulatory settings, leverage electronic health records (EHR) in an effort to inform and engage patients [9, 10]. Until recently, little attention had been given to using portals as a tool for hospitalized patients. Several small studies have evaluated the use of tablet computers with mobile patient portal applications designed for hospitalized patients [11–14]. Although results indicate patients are generally enthusiastic about such applications, the impact on patient comprehension, engagement, and clinical outcomes is largely unknown. Our research team recently developed and tested a hospital-based mobile patient portal which included information about the medical team, scheduled tests and procedures, and a list of active medications [15]. While the portal improved patients’ knowledge of physician team members, we found no effect on patient knowledge of the care plan or activation. Importantly, patient use of the portal was highly variable. We hypothesized that limited use of the portal by some patients may have contributed to these findings. Thus, the overarching goal of this study was to identify barriers to portal use and solicit user input to inform the design of future versions of a hospital-based patient portal.

The specific objectives of this study were to explore: (1) patient perspectives regarding portal content and features perceived to be most beneficial, (2) patient barriers to portal use and recommendation for improvement, and (3) healthcare provider perspectives regarding both benefits and challenges that a hospital-based patient portal may pose.

## Methods

### Setting

This qualitative study was conducted at Northwestern Memorial Hospital (NMH), a large academic hospital in Chicago, Illinois. Participants included patients, physicians, and nurses on two general medical services: a resident-covered teaching service and a non-teaching hospitalist service. General medical patients were admitted to one of these two services based on bed availability. The Northwestern University Institutional Review Board approved the study.

### The mobile patient portal application

The development of the portal application has been previously described [15]. Briefly, the portal was organized into pages reflecting general patient information, the care team, the medication list, and the agenda for the day (See Additional file 1). General patient information included allergies, the problem list, and the name of the patient’s primary care physician. The care team page included the names and pictures of the nurse and primary service physicians, and a description of their roles. The medication list populated from active medication orders and provided hyperlinks to Lexicomp® patient drug information. In an effort to minimize the effect of this initial version of the portal on provider workflow, we did not provide patients with access to laboratory or radiology reports and did not allow messaging with providers. Therefore, providers did not use the portal as part of their work activities during the study. We installed the portal application on 10 Apple iPad tablet computers (Retina display Wi-Fi 16GB – 4th generation) along with a Mobile Device Management Solution by Symantec, which restricted tablet computer functionality to use only on the secured hospital network. In addition to the portal, tablets included links to a web browser (Safari), entertainment (e.g., Netflix), games (e.g., Candy Crush, solitaire), and social media (e.g., Facebook). We created unique passwords for each tablet computer prior to distribution, and encouraged patients to update the password. Following patient discharge, we collected, sanitized, and reset the password on each tablet prior to distributing to another patient.

### Patient enrollment and data collection

Each weekday morning from February 1 through March 30, 2015, a research coordinator (AK) randomly selected patients newly admitted to either the resident-covered teaching service or non-teaching hospitalist service and offered them use of a tablet computer with portal access for the duration of their hospital stay. We excluded non-English speaking patients and those not oriented to person, place and time. Patients were also excluded if they had been transferred from another service or had physical or visual impairments that impeded use of a tablet computer. After obtaining written informed consent, the research coordinator briefly instructed patients on the use of the tablet and portal application, provided contact information for one-on-one technical support, and scheduled mutually convenient times to conduct semi-structured interviews. The research coordinator conducted interviews on patients’ third or fourth hospital day to allow sufficient time for patients to use the portal (i.e., 2–3 days) and maximize the efficiency of data collection. Semi-structured interviews of patients focused both on their experiences with, and recommendations for changes to, the portal (See Patient Interview Questions in Additional file 2). Patients did not receive an incentive for participating in the study. Interviews were audio recorded for subsequent transcription and coding.

### Provider enrollment and data collection

We obtained lists of eligible providers from clinical schedules and clinical leaders. Eligible physicians and nurses received an email introducing the study and asking for their participation. We also recruited physicians and nurses at regularly scheduled meetings (e.g., residency program meetings for residents and divisional meetings for hospitalists). We used these strategies to identify participants until we had a sufficient number (6–8) for each focus group. We conducted three focus groups of healthcare providers; one consisting of nurses, one with internal medicine residents, and one with hospitalists. Though all healthcare providers had cared for patients using the portal, many had limited direct interaction with the portal. As such, we began each discussion with a brief description of portal features and provided a tablet computer with the portal and access to simulated patient information. After obtaining consent, the focus group moderator (AK) asked participants to discuss their experiences caring for patients with the portal and the benefits and challenges related to the portal (See Healthcare Provider Focus Group Questions in Additional file 2). We also asked providers how potential new portal features, like access to results and messaging, might affect patients and providers. We provided lunch, but no other incentives for focus group participation. All focus group discussions were audio recorded.

### Data analysis

Digital audio recordings from interviews and focus groups were transcribed verbatim and participant identifiers removed to maintain confidentiality. Two investigators (AK and LSO) independently coded the transcribed text using constant comparative techniques to identify codes and group codes into overarching themes [16]. A third investigator (KJO) then reviewed themes with the two coders and coding differences were resolved through consensus. We used MAXQDA version 11 to manage the coding process. Recruitment and analysis continued until thematic saturation was achieved.

## Results

### Participants

Overall, 91 patients were approached to receive a tablet computer with access to the portal during their hospitalization. Forty-two patients were excluded because of disorientation, ten were non-English speaking, and eight had visual and/or physical impairment. Seven patients declined to participate and six were discharged before completion of the interview. Eighteen patients completed semi-structured interviews and 21 healthcare providers (six hospitalists, eight resident physicians, and seven nurses) participated in the three focus groups. Characteristics of participants are shown in Tables 1 and 2.

**Table 1:** Patient characteristics

Characteristic, *n* (%)	*N* = 18
Mean age (SD)	40.5 ± 11.7
Women	13 (72.2)
Nonwhite	8 (44.4)
Case mix	
Diseases of the blood	4 (22.2)
Diseases of the digestive system	2 (11.1)
Symptoms, signs, and ill defined conditions	2 (11.1)
Diseases of the genitourinary system	2 (11.1)
Diseases of the respiratory system	2 (11.1)
Other	6 (33.3)
Mean MS DRG weight (SD)^a^	1.3 (0.7)
Mean Elixhauser score (SD)	6.9 (9.5)
Mean length of stay (SD)	4.5 (4.1)

^a^ *MS DRG* Medicare Severity Diagnosis Related Group

**Table 2:** Healthcare provider characteristics

	Hospitalist physicians (*n* = 6)	Resident physicians (*n* = 8)	Nurses (*n* = 7)
Mean age (SD)	39.0 (7.1)	29.8 (2.1)	36.6 (14.7)
Female, *n* (%)	5 (83.3)	3 (37.5)	6 (85.7)
Nonwhite race, *n* (%)	2 (33.3)	3 (37.5)	0 (0)
Mean years at hospital (SD)	2.8 (2.2)	2.0 (0.9)	11.9 (13.7)

### Thematic domains

We identified five emergent themes from patient interviews and provider focus groups. Both patients and providers identified benefits and challenges related to information provided and opportunities and challenges related to patient-provider communication. Emergent themes unique to patients included use of the tablet computer as an entertainment device and technical challenges. Providers uniquely identified issues related to the portal’s impact on workflow.

### Patient semi-structured interview results

#### Benefits and challenges related to the information provided by the portal

Patients reported that their primary method for obtaining information about their care was through verbal discussion with physicians during rounds. Many patients appreciated the additional information provided by the portal and generally found it useful as illustrated by a patient who stated *“I mean the more information you have; the better you are when you’re in the hospital. I like the fact that it [the portal] gives you all the information about my meds and my doctors and stuff like that”* (Patient 5)*.* Regarding specific information provided, patients appreciated the names, role descriptions, and pictures of team members. Patients also expressed favorable opinions about the medication information provided, but identified opportunities for improving content, including showing the time of the last dose administered for “as needed” medications and displaying both brand and generic names of medications. One patient explained *“I take the ones at home that are brand names. What they’re giving me here [in the portal] is a generic name”* (Patient 14).

Many patients expressed a desire for more information, and specifically to view results via the portal. One patient explained *“like, when they take the blood, I’d like to know the results”* (Patient 15). Another commented *“I wish it connected to results of blood tests and stuff…like when they say my liver enzymes were elevated, how much? I get that they’re elevated, but like, significantly? Give me the numbers because I want to know”* (Patient 6).

#### Opportunities and challenges related to patient-provider communication

Several patients described situations in which they had a question shortly after physicians’ daily rounds had occurred and identified the portal as a potential solution. One patient commented *“You come up with a question ten minutes after they leave”* (Patient 1). The same patient suggested *“I think that if there was a place that I could type in my questions, like even if it’s just to save them for later. If they could see them, I don’t even know if that matters.”*

#### Use of the tablet computer as entertainment

Many patients described frequent use of the games and other non-clinical applications accessible via the tablet. One patient stated *“I probably played the games for a couple-two or three hours yesterday and today a bit…It was nice to have something to do besides just watch TV”* (Patient 4). Furthermore, using the tablet for entertainment seemed to assist with coping and served as a positive distraction for some patients experiencing acute exacerbations of chronic illness. One patient discussed his struggles with sickle cell anemia, and keeping a positive outlook: *“It’s rough. I mean, there’s almost as comparing it to one who would have cancer. It’s a day by day thing, so just got to stay, stay positive, have your faith, and, and you deal with it…[the tablet] was something that I started to explore and use game-wise or, or for music. Keep my mind busy, so yeah, it, it came in handy”* (Patient 2).

#### Technical challenges

Patients generally found the tablet and portal easy to use, but some patients identified technical issues which served as barriers to using the portal. For example, one patient commented that he was able to see all information on the page when the tablet was in landscape orientation, but needed to swipe left to see information when in portrait orientation. Another patient experienced difficulty after forgetting his password, stating *“I ended up locking myself out of it and then I couldn’t remember the password. I could’ve called the number, I know, and asked for it”* (Patient 16). Another patient described physical difficulty using the tablet, explaining *“the iPad for me, especially with a lung condition right now, was too heavy to hold”* (Patient 6).

### Provider focus group results

#### Benefits and challenges related to the information provided by the portal

Healthcare providers felt the information provided by the portal helped facilitate patient engagement in care and the identification of errors. For example, one resident noted *“the experience that I’ve had with it is a patient as well as a family member mentioning that medications, saying that they hadn’t received the medication yet or the timing was slightly off [compared to how] they took it home”* (Resident 3). Providers suggested enhancements to portal information, like showing the purpose or class of medications: *“I like that the instructions are given in patient-friendly language, but it doesn’t give you what the medication type is. Who knows what Piperacillin is? But if it was like Piperacillin – this is an antibiotic; or hydrochlorothiazide – blood pressure medicine”* (Hospitalist 2). Regarding the possible provision of results to patients, providers worried about the high volume of tests performed in the hospital setting, the high percentage of abnormal test results, and patients’ ability to interpret results in the context of their acute illness:

Resident 2: *“Almost everyone that’s in the hospital has abnormal labs every day pretty much.”*

Hospitalist 1: *“I feel like on any CAT scan, there’s 30 different incidental findings that’s just going to add confusion, questions, things that a patient does not need to worry about.”*

Nurse 4: *“Even sometimes I read some of these reports; I’m like what is this word?”*

Many healthcare providers also expressed concerns related to the procedure for releasing certain types of information to patients via the patient portal. Physicians, especially, worried that sensitive information related to new diagnoses or worsened condition risked being conveyed via the portal rather than in-person by the appropriate clinician. Physicians seemed to understand that a variety of options (e.g., manual release by clinician, automated timed release) exist for the selection and timing of result release and that each option had advantages and disadvantages:

Hospitalist 2: *“One worry I have is if the patient gets their test results maybe before we've seen them.”*

Resident 7: *“If you are releasing labs – and it’s not an auto fill thing, you’re going to have to pick and choose what labs and, like at some point today I’m going to sit down and just go through my patient list and be like, I think this person should see these labs and I’ll write a little blurb about why it’s important.”*

Healthcare providers also pointed out that many hospitalized patients rely on family members and loved ones to serve as surrogate decision makers. Providers suggested that remote access to patients’ designees could serve as an important tool to inform surrogates who are often unable to be physically present during physician rounds. One hospitalist explained: *“You know, there are surrogates who really are the ones who can get all the information way more than the patient…maybe they could access it online – you know if they are at work during the day and they want to know what their loved one’s test results were”* (Hospitalist 5).

#### Opportunities and challenges related to patient-provider communication

Many healthcare providers felt the portal enhanced the quality of discussions with patients during rounds. One hospitalist commented: *“They already have the information as far as their meds or their scheduling and they ask you more pertinent questions with respect to those, like ‘What is this medication?’ or ‘Why am I taking it this many times?’”* (Hospitalist 3). Similar to the comments from patients, providers suggested that patients should have the ability to take notes and/or write questions in the portal. One hospitalist said: *“I think a nice feature could be some way for the patient to record the questions they might have throughout the day…if they have a question or even if there’s a way to flag a result that could be collated into a list of questions for the provider when they come in”* (Hospitalist 2).

Healthcare providers expressed concerns about the potential to use the portal for other forms of communication, like two-way messaging. Physicians felt messaging had the potential to be used too frequently, inappropriately to communicate urgent issues, and might adversely affect their relationship with patients:

Hospitalist 1: *“I imagine someone messaging you saying, ‘I'm having chest pain.’”*

Hospitalist 2: *“This potentially adds a barrier to communication that if we are defaulting to just texting or messaging our patient back, that eliminates our face to face interaction which I think has a lot of healing also.”*

#### Impact on providers’ workflow

Healthcare providers perceived minimal impact on their workflow during the study, but expressed concern over what effect the patient portal would have on their daily routine and workload with additional features. Specifically, providers mentioned the likelihood of the portal to generate additional questions if test results were made available to patients. Although it was recognized that additional questions might result in a more informed patient, concerns were raised regarding the additional time that would be needed to answer what could be a significantly increased number of questions:

Hospitalist 1: *“I just imagine a lot more discussion which would be great in the end. I think it will help patient care but I imagine it will be a lot more time consuming.”*

Resident 7: *“I just feel like on rounds, explaining every lab test that’s a little bit off, it can take forever. And as it is, we’re like, pressed for time.”*

Nurses worried about their responsibility for iPads if a larger scale implementation were to occur. One nurse commented *“I mean we can’t keep track of phones, let alone iPads”* (Nurse 7).

## Discussion

We found that participants generally felt the portal was useful as a tool to inform and engage patients. However, patients and healthcare providers readily identified opportunities for improvement. Several themes emerged which reveal opportunities to enhance the design of portals intended for hospitalized patients.

Regarding portal content, both patients and providers described the utility of including information about current medications and several suggested enhancements to the portal such as including timing of the last dose for “as needed” medications, use of both generic and brand names, and indicating the class or purpose of medications. Regarding potential additional features, patients expressed interest in test results. This finding is consistent with research by Dykes, Dalal, and colleagues demonstrating that hospitalized patients have a strong interest in all types of test results [14, 17]. Importantly, healthcare providers in our study worried that, should results be made available to patients, the large volume, high percentage of abnormal results, and complex medical terminology could cause anxiety and confusion for patients. Importantly, studies of outpatient portals have found that few patients report increased anxiety or difficulty understanding the clinical information [18–20]. Less is known about providing access to clinical information for hospitalized patients. Prey and colleagues published a study in which they provided hospitalized patients with paper copies of their medical records including physicians’ progress notes, laboratory test results, radiology reports, and operative reports [21]. Patients perceived the information as highly useful even if they did not fully understand complex medical terms. Pell and colleagues also recently published a study in which they provided tablet computers to hospitalized patients, allowing access to medication schedules, laboratory results, and plain radiography results to patients on a medical unit [12]. Although comprehension was not objectively assessed, use of the portal did not increase patient reported anxiety or confusion. These studies suggest that clinicians’ concerns may be unfounded, but further research is needed to objectively assess the impact of providing clinical information via a portal on the levels of anxiety and comprehension among hospitalized patients.

Healthcare providers expressed a desire to control which results were released to patients, the timing of release, and to annotate the results. However, providers also identified the potential for such manual release to increase their workload. Overall, these findings highlight the need for careful decisions related to type of results to release, as well as the timing, rules, and format of display. While principles of transparency, patient empowerment, and shared decision making would support immediate release of results, the most pragmatic approach likely entails pre-selecting certain results for timed automated release. For example, an identified rubric could require that all basic chemistry and blood count results finalized before 7 AM as well as plain radiograph, CT, and MRI results finalized before midnight be automatically released at noon, whereas pathology reports and sensitive laboratory results (e.g., HIV, CD4 counts) might be communicated only through verbal discussion.

We found that both patients and providers were interested in giving patients the ability to record notes and questions. More advanced communication options, like two-way messaging via the portal, were strongly opposed by healthcare providers. Physicians feared that patients would send more messages than they could manage, have unrealistic expectations regarding timeliness of response, inappropriately use messaging for urgent clinical matters, and that messaging might damage the patient-physician relationship. Although studies of ambulatory based portals have found a high level of patient satisfaction with secure messaging, data on physicians’ perceptions is lacking [10, 22, 23]. Dalal and colleagues recently published a study of a hospital-based patient portal in which two-way messaging was available [14]. The volume of messages was fairly low (~1.8 messages per patient), which they attributed to efforts to set expectations at the time of enrollment and lack of timely responses from providers.

Recognizing the importance of surrogate decision makers and that surrogates are often unable to be present during physician rounds, healthcare providers suggested that family members and loved ones be given access to the patient portal. This recommendation is supported by a recent study by Torke and colleagues who found that surrogates were involved in decision making for nearly half of hospitalized older adults [24]. Providing remote access to surrogates would require specific steps at both a local and national level [25]. At the local level, hospitals need to develop policies allowing patients to appoint designees and strong authentication procedures using a unique login and password for each designee. At a higher level, federal support is needed to develop standards in this area and for research to understand how to best provide remote access.

The game and entertainment applications on the tablets were extremely popular among patients. Several patients described how use of games helped them cope with their illness. This finding is unique among studies of hospital-based patient portals, but consistent with prior research showing that video games can serve as a positive distraction and a complement to other symptom management strategies [26, 27]. In light of the variable use found in our initial hospital-based portal study and generally low adoption of ambulatory-based portals [11, 15, 28], patients’ interest in games and entertainment could also be leveraged to promote use of the portal and enhance engagement in care. For example, patients could be asked to review and confirm understanding of their current medication list prior to being given access to additional game applications. Gamification of the portal itself, may also promote use [29]. A recent systematic review published by Otte-Trojel and colleagues on the development of patient portals found that few studies had evaluated the use of promotional initiatives incorporated into portal design to attract patient attention and encourage use [30].

Patients generally found the portal application easy to use. Several patients identified opportunities to improve the portal interface, all of which appear easily achievable. Some patients identified barriers related to the tablet itself, highlighting the need to provide devices that accommodate patients with physical and visual impairment.

Our study has several limitations. We conducted a qualitative evaluation of a custom designed, mobile patient portal application at a single site. Though our sample size was relatively small, we achieved thematic saturation. Prior research has shown that saturation often occurs in interview studies within the first 12 interviews and basic elements for themes may be present as early as six interviews [31]. Our portal was designed for hospitalized patients and lacked content and features frequently available in ambulatory-based portals. Our overarching goal was to identify barriers to portal use and solicit user input to inform the design of future versions of a hospital-based patient portal. Though patient characteristics and provider workflow may vary, we believe our findings are generalizable to other hospital settings. Importantly, our findings can help inform a range of decisions during implementation and adaptation of EHR vendor developed patient portals for use in hospital settings (e.g., Epic MyChart Bedside). Finally, a single individual (AK) conducted the interviews, moderated the focus groups, and served as one of the two primary analysts. Though relatively common in qualitative research, this arrangement may have increased the potential for the researcher to become anchored prematurely to initial impressions.

## Conclusion

In conclusion, our assessment of patient, physician, and nurse perceptions of a hospital-based patient portal revealed important findings that should inform design decisions to promote use and foster engagement. Optimizing a hospital-based patient portal will require careful attention to type, timing and format of information provided, as well as the impact on provider workflow and patient-provider communication.

## Additional files

Additional file 1: Screenshots of Patient Portal: this appendix provides an example of portal content and features. (PDF 2005 kb) Additional file 2: Patient Interview and Provider Focus Group Questions: this appendix provides the questions used during semi-structured interviews of patients and focus groups of healthcare providers. (DOCX 15 kb)

## Abbreviations

EHR: Electronic health record
NMH: Northwestern Memorial Hospital
