# Supplementary material for: Patients’ and healthcare providers’ perceptions of a mobile portal application for hospitalized patients
Source: BMC Med Inform Decis Mak. 2016 Sep 21;16:123. doi: 10.1186/s12911-016-0363-7 (PMC5031299; doi:10.1186/s12911-016-0363-7)
Supplement: Additional file 1: — Screenshots of Patient Portal: this appendix provides an example of portal content and features. (PDF 2005 kb) [file 12911_2016_363_MOESM1_ESM.pdf]

Login With PatientConnect | Login or Sign Up With MyChart | Login With RelayHealth | Login With athenahealth

Good Morning John Doe

iPad Instructions   Feedback

Home

My Care

Hospital

Welcome to Northwestern Memorial Hospital.

Today's Schedule

8:00 AM	9:30 AM	Breakfast
9:00 AM	11:30 AM	Physician Rounding
11:30 AM	1:00 PM	Lunch
5:00 PM	7:00 PM	Dinner
Upcoming		Consistent Carbohydrate (ADA Diet)

See More Events...

Currently
61°F
Overcast

Tomorrow
High 64°F
Low 54°F

forecast provided by WEATHER UNDERGROUND

Google News

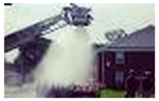

SO SAD: Fire Captain, Injured During Ice Bucket Challenge, Dead One Month ... - Global Grind
Google - 10 hours ago

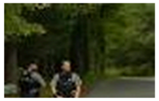

Police find weapon belonging to cop killing suspect - USA TODAY
Google - 2 hours ago

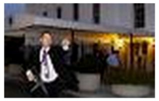

Secret Service reviews White House security after fence-jumper enters mansion - Washington Post
Google - 2 hours ago

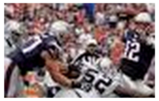

Purdy: Raiders are what we thought they were - San Jose Mercury News
Google - 2 hours ago

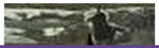

New Afghan president, but vote [illegible]

powered by FeedGrabbr

Access to NMHC resources through this system is subject to the terms of the NORTHWESTERN MEMORIAL HEALTHCARE INFORMATION ACCESS AND CONFIDENTIALITY AGREEMENT. This system may be accessed and used by authorized personnel only. Authorized users may only perform authorized activities and may not exceed the limits of such authorization. Disclosure of information found in this system for any unauthorized use is strictly prohibited. All activities on this system are subject to monitoring.

John Doe’s Profile

Home

My Care

Hospital

Allergies

Advil	Active

Active Conditions

PYELONEPHRITIS, UNSPECIFIED	2014-09-20
HYPERTENSION	2014-09-20

My Profile

My Care Team

My Medications

My Agenda

Personal Information

**Primary Care Provider:**	DAVID LIEBOVITZ
**Unit:** Feinberg 13 E, 1313	
**Medical Service:** GENERAL MEDICINE	
**Email:**	

John Doe’s Care Team

Home

My Care

Hospital

VERMYLEN, MD, JULIA L.

Attending Doctor
The attending is the doctor in charge of all the care you receive; they will work with you and the rest of the healthcare team to identify the best treatment plans and goals.

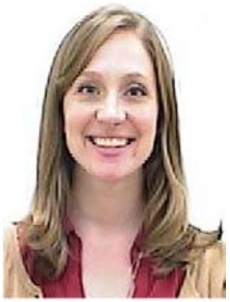

Rogalla, Heidi

Nurse
Nurses perform routine patient care activities and give updates to the healthcare team about how a patient is feeling. Let them know if you need anything.

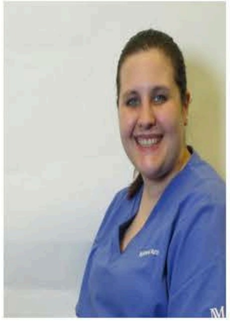

KAMATH, SUNEEL

Resident
Residents are doctors that are training to become attending physicians; they can do everything the attending can, but will talk to the attending before any changes in your care are made.

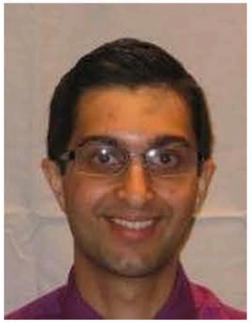

WHITSETT, MAUREEN

Intern
Interns are doctors in their first year out of medical school; they are training to become residents and perform many of the same duties.

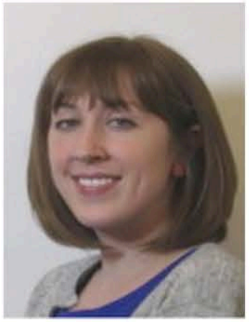

My Profile

My Care Team

My Medications

My Agenda

Clinicians shown gave permission to have their photographs and information displayed

John Doe's Medications

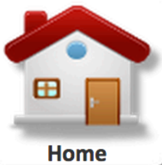

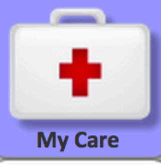

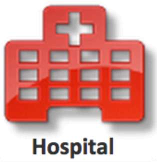

Scheduled Medications

**Hydrochlorothiazide 25mg Tab**<br>Route:Oral,Frequency:Daily<br>Last Administered:09-21-2014 12:59	Click for Info: UpToDate
**Lansoprazole 30mg Cap DR**<br>Route:Oral,Frequency:Daily (7 AM)<br>Last Administered:09-21-2014 12:59	Click for Info: UpToDate
**Lisinopril 20mg Tab**<br>Route:Oral,Frequency:Daily<br>Last Administered:09-21-2014 09:50	Click for Info: UpToDate
**Nystatin 100,000unit/g Powder 15g**<br>Route:Topical,Frequency:Twice per Day<br>Last Administered:09-21-2014 09:50	Click for Info: UpToDate
**Piperacillin/Tazobactam + Sodium Chloride 0.9% 100 mL**<br>Route:IVPB,Frequency:Every 12 Hours<br>Last Administered:09-21-2014 16:59	Click for Info: UpToDate

Take as Needed

**Fluticasone Propionate 0.05% Nasal Spray 16g**<br>Route:Each Nostril	Click for Info: UpToDate
**Acetaminophen 325mg Tab**<br>Route:Oral	Click for Info: UpToDate
**Insulin lispro (rDNA) (HumaLOG) 100unit/mL Inj MDVL 3mL**<br>Route:Under the Skin	Click for Info: UpToDate
**Dextrose 50% Inj LIFESH 50mL**<br>Route:IV Push	Click for Info: UpToDate

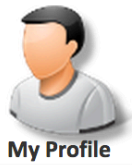

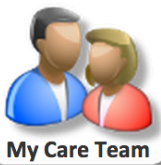

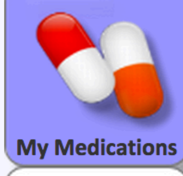

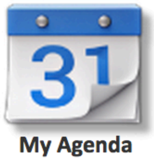

Access to NMHC resources through this system is subject to the terms of the NORTHWESTERN MEMORIAL HEALTHCARE INFORMATION ACCESS AND CONFIDENTIALITY AGREEMENT. This system may be accessed and used by authorized personnel only. Authorized users may only perform authorized activities and may not exceed the limits of such authorization. Disclosure of information found in this system for any unauthorized use is strictly prohibited. All activities on this system are subject to monitoring.
