# Supplementary material for: Patients’ and healthcare providers’ perceptions of a mobile portal application for hospitalized patients
Source: BMC Med Inform Decis Mak. 2016 Sep 21;16:123. doi: 10.1186/s12911-016-0363-7 (PMC5031299; doi:10.1186/s12911-016-0363-7)
Supplement: Additional file 2: — Patient Interview and Provider Focus Group Questions: this appendix provides the questions used during semi-structured interviews of patients and focus groups of healthcare providers. (DOCX 15 kb) [file 12911_2016_363_MOESM2_ESM.docx]

**Additional file 2 – Patient Interview and Provider Focus Group Questions**

**Patient interview Questions**

We are interested in your experience with the patient portal.

1. Tell me about your experience with the patient portal.
2. What did you find most helpful?
3. What was least helpful?
4. What challenges did you experience in using the portal?
5. What changes would you make the portal?

**Healthcare Provider Focus Group Questions**

We are interested in learning about the patient portal from the providers’ perspective.

1. Tell me about your experiences caring for patients who have the patient portal.
2. How might the portal help healthcare providers?
3. How might the portal create challenges for healthcare providers?

We would like to improve the portal and would like your advice. Potential improvements might include additional information, like lab results, and radiology reports.

1. How might features like these help healthcare providers?
2. How might features likes these create challenges for healthcare providers?

An additional feature might include two-way messaging between patients and their providers.

1. How might a feature like this help healthcare providers?
2. How might a feature like this create challenges for healthcare providers?
